# Elevated Cellular PD1/PD-L1 Expression Confers Acquired Resistance to Cisplatin in Small Cell Lung Cancer Cells

**DOI:** 10.1371/journal.pone.0162925

**Published:** 2016-09-09

**Authors:** Fei Yan, Jiuxia Pang, Yong Peng, Julian R. Molina, Ping Yang, Shujun Liu

**Affiliations:** 1 The Hormel Institute, University of Minnesota, 801 16th Avenue NE, Austin, Minnesota, 55912, United States of America; 2 Department of Thoracic Surgery, State Key Laboratory of Biotherapy, West China Hospital, Sichuan University /Collaborative Innovation Center of Biotherapy, Chengdu, 610041, China; 3 Department of Medical Oncology, Mayo Clinic, 200 1st Street SW, Rochester, Minnesota, 55905, United States of America; 4 Division of Epidemiology, Mayo Clinic, 200 1st Street SW, Rochester, Minnesota, 55905, United States of America; Universidad de Chile, CHILE

## Abstract

Although small cell lung cancer (SCLC) is highly responsive to chemotherapies (e.g., cisplatin-etoposide doublet), virtually almost all responsive SCLC patients experience disease recurrence characterized by drug resistance. The mechanisms underlying cisplatin resistance remain elusive. Here we report that cell-intrinsic expression of PD1 and PD-L1, two immune checkpoints, is required for sustained expansion of SCLC cells under cisplatin selection. Indeed, PD1 and PD-L1 were expressed at a higher level in lung cancer cell lines, tumor tissues, and importantly, in SCLC cells resistant to cisplatin (H69R, H82R), when compared to respective controls. Genetic abrogation of PD1 and PD-L1 in H69R and H82R cells decreased their proliferation rate, and restored their sensitivity to cisplatin. Mechanistically, PD-L1 upregulation in H69R and H82R cells was attributed to the overexpression of DNA methyltransferase 1 (DNMT1) or receptor tyrosine kinase KIT, as knockdown of DNMT1 or KIT in H69R and H82R cells led to PD-L1 downregulation. Consequently, combined knockdown of PD-L1 with KIT or DNMT1 resulted in more pronounced inhibition of H69R and H82R cell growth. Thus, cell intrinsic PD1/PD-L1 signaling may be a predictor for poor efficacy of cisplatin treatment, and targeting the cellular PD1/PD-L1 axis may improve chemosensitization of aggressive SCLC.

## Introduction

Small cell lung cancer (SCLC) represents ~15% of all lung cancer cases and is one of the most lethal malignancies [1, 2]. For decades, etoposide and platinum (EP doublet) have represented the generally accepted standard first-line therapy [3–5]. SCLC is usually very chemosensitive with response rates up to 80% [6, 7]. However, almost all patients have disease progression within months post therapy. Recurrent SCLC is then more aggressive, with less response to therapy compared to primary disease, for instance, 3–25% for topotecan, a topoisomerase I inhibitor [8]. There are no effective treatment regimens for patients whose disease has progressed after first- and second-line therapy. While many resistance mechanisms have been described and multiple regimens targeting such resistant factors have been in clinical trials, SCLC prognosis remains one of the worst in all malignancies, indicating the existence of additional signaling networks in regulating SCLC cell fate in response to chemotherapy.

Cisplatin, a platinum-derivative agent, is one of the most potent antitumor agents, displaying clinical activity against a wide variety of solid tumors, including SCLC [9]. Its best understood mode of actions involves the generation of DNA lesions followed by the activation of several signal transduction pathways, including ATR, p53, p73 and MAPK, which leads to cell apoptosis [10–12]. Despite a consistent rate of initial responses, disease progression almost invariably occurs and chemoresistance rapidly emerges [13, 14]. In the past decades, tremendous efforts have been made in understanding and fighting chemoresistance; several mechanisms that account for the cisplatin-resistant phenotype of tumor cells have been described [15–17]. However, the therapeutic regimens developed from these reported mechanisms have failed to achieve improved outcomes in SCLC patients, indicating the need of new treatment options that are built on new mechanistic findings.

The programmed cell death 1 (PD1) is a prominent checkpoint receptor. Upon engagement by its ligands, PD-L1/PD-L2, in the tumor microenvironment, PD1 dampens T effector functions, thus protecting cancer cells from immune-mediated rejection [18–21]. The PD1/PD-L1 signaling also has cell-intrinsic functions in certain types of mouse and human tumors through modulating downstream effectors of mTOR signaling [22, 23]. As it boosts cancer growth and promotes tumorigenesis, a number of antibody-based therapeutics targeting the PD1/PD-L1 axis have entered clinical trials. Notably, PD1 blockade yielded clinical activity in patients with immunogenic cancers [24] as well as those with lesser immunogenic cancers [23]. However, many tumors are refractory to treatment with single antibody and the adverse effects occur through nonspecific immunologic activation [22]. Further, the doses of PD1 agents greater than 1 mg/kg seem not to increase efficacy. These pitfalls call for combination approaches to enhance the therapeutic effectiveness of PD1/PD-L1 blocking agents. In the present study, we generated SCLC cells H69R and H82R resistant to cisplatin, and present evidence that PD1/PD-L1 are expressed at a higher level in resistant versus parental cells. We show that PD-L1 upregulation in resistant cells results from overexpression of DNMT1 or KIT, and abrogation of PD-L1 restores cisplatin resistance. Further, co-depletion of PD-L1 with DNMT1 or KIT led to more pronounced inhibition of resistant SCLC cell growth. These findings shed a light on the cisplatin resistance mechanisms and highlight PD1/PD-L1 signaling as a potential therapeutic target in refractory SCLC patients.

## Results

### The expression of PD1 and PD-L1 is elevated in lung cancer cells

**Fig 1:**
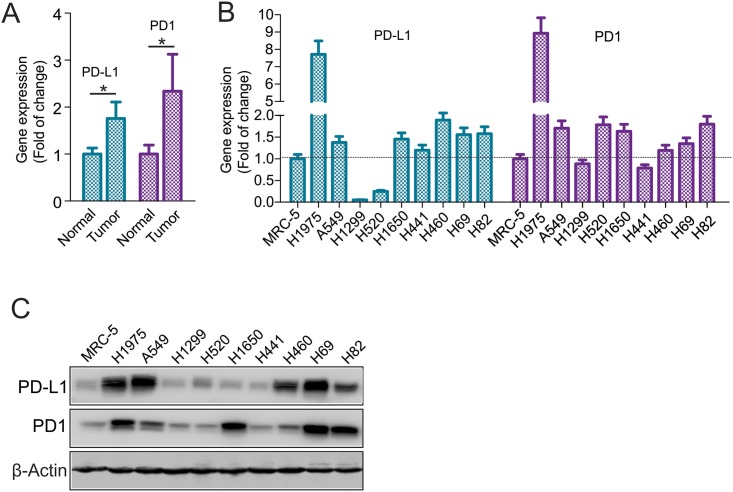
PD1 and PD-L1 are highly expressed in lung cancer cells. A, qPCR measuring PD1 and PD-L1 expression in fresh-frozen human lung normal or cancer tissues (n = 30/group). Data represent the mean + SD, *P < 0.05. B and C, qPCR (B) and Western blot (C) analysis assessing PD1 and PD-L1 expression in human normal and lung cancer cell lines. Data represent three independent experiments.

Although main studies regarding PD1/PD-L1 focus on the immune response [18, 19], certain reports showed that PD1/PD-L1 are highly expressed in human and mouse tumors [22]. To this end, we carried out quantitative PCR (qPCR) for mRNA from fresh-frozen non-small cell lung cancer (NSCLC) patient tissues and found that the levels of both PD1 and PD-L1 are higher in fresh-frozen patient tumors compared to the normal adjacent tissues (n = 30) (Fig 1A). Consistently, the results of qPCR (Fig 1B) and Western blot (Fig 1C) revealed that, compared to normal lung fibroblast MRC-5, the NSCLC and SCLC cancer cell lines, H1975, A549, H1650, H460, H69 and H82, had higher expression of PD1 and PD-L1, in agreement with previous findings that PD1 and PD-L1 are upregulated in lung cancer cells [25–27]. These results support a notion that PD1 and PD-L1 could have cellular functions in lung cancer cell growth.

### Chronic exposure to cisplatin causes upregulation of PD1/PD-L1

**Fig 2:**
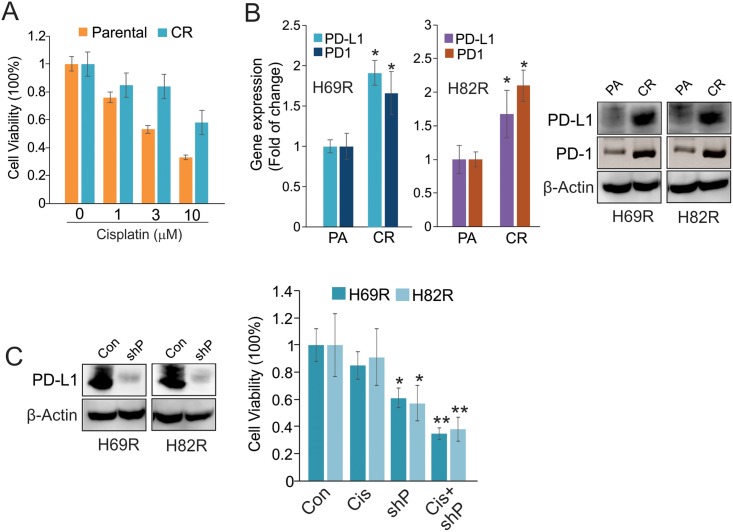
PD1/PD-L1 signaling sustains the survival and proliferation of cisplatin resistant cells. A, Resistant and parental H69 cells were treated with indicated doses of cisplatin and subjected to CCK8 assays. Note: CR, Cisplatin resistance. B, The H69R and H82R cells were growing in drug-free medium for 72 hours. The qPCR or Western blot was used to measure the RNA (left panel) or protein (right panel) expression of PD1 or PD-L1. Data represent three independent experiments, and are the mean ±SD, *P < 0.05. Note: PA, Parental; CR, Cisplatin resistance. C, H69R and H82R cells were transfected with PD-L1 shRNA or control vectors for 24 hours, and then exposed to 3 μM of cisplatin for additional 24 hours. Western blot (left panel) was used to measure PD-L1 protein expression, but CCK8 assays for the cell proliferation. Note: Con, Control vectors; shP, PD-L1 shRNA; Cis, Cisplatin. In CCK8 assays, the experiments are done two times independently with 8 replicates. *P < 0.05, **P < 0.01.

To delineate the molecular mechanisms underlying cisplatin resistance, SCLC cells H69 and H82 were exposed to increasing concentrations of cisplatin (0.1, 0.3, 1, 3 μM) for 6–8 weeks, until they could be cultured in 3 μM cisplatin. Cells cultured in parallel without drugs served as parental sensitive controls. To characterize drug-resistant phenotypes, we measured the proliferation rate of H69 resistant versus parental cells upon transient exposure to cisplatin up to 10 μM. While the proliferation of parental cells was dose-dependently inhibited, cisplatin had a minimal effect on H69R cells (Fig 2A). When H69R and H82R cells were growing in drug-free medium for 72 hours, the results of qPCR and Western blot revealed an upregulation of PD1/PD-L1 in resistant cells compared to parental controls (Fig 2B). These findings indicate that PD1/PD-L1 deregulation plays a role in the survival and proliferation of cisplatin resistant cells. To address the cellular functions of PD-1/PD-L1 signaling in cisplatin resistance, we transfected H69R and H82R cells with PD-L1 shRNA or scramble vectors for 24 hours, and then incubated them with cisplatin (3 μM) for additional 24 hours. The CCK8 assays revealed that, compared to scramble vector, PD-L1 depletion (Fig 2C, left panel) induced more cell growth arrest in the presence of cisplatin (Fig 2C, right panel), suggesting that PD-L1 abrogation restored the sensitivity of H69R and H82R to cisplatin treatment. Thus, PD-L1 upregulation might be required for H69R and H82R to survive under cisplatin selection.

### Expression of PD-L1 is attributed to DNMT1 or KIT upregulation in H69R and H82R cells

**Fig 3:**
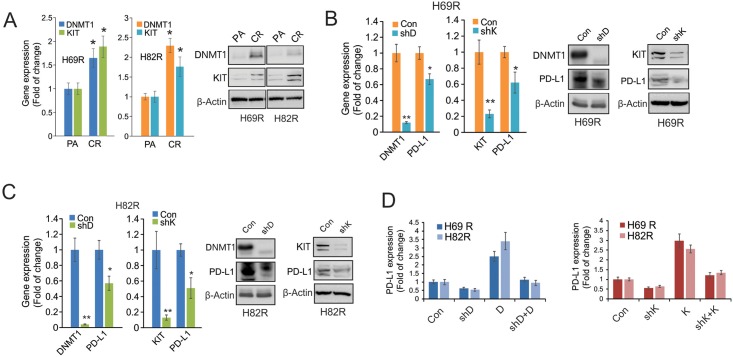
DNMT1 and KIT are the upstream regulators of PD-L1 signaling in cisplatin resistant cells. A, The H69R and H82R cells were growing in drug-free medium for 72 hours. The qPCR (left panel) or Western blot (right panel) analysis was used to assess the expression of DNMT1 and KIT. B and C, H69R (B) and H82R (C) cells were transfected with DNMT1 or KIT shRNA for 48 hours and subjected to the qPCR (left panel) or Western blot (right panel) analysis for the expression of indicated genes. D, H69R and H82R cells were transfected with DNMT1 or KIT shRNA for 24 hours, and then DNMT1 or KIT expression vectors were introduced for additional 24 hours. The expression of PD-L1 was determined by qPCR. Note: PA, Parental; CR, Cisplatin resistance; Con, Control vectors; shD, DNMT1 shRNA; shK, KIT shRNA; D, DNMT1 expression vectors; K, KIT expression vectors. Data represent three independent experiments, and are the mean ± SD, *P < 0.05, ** *P* < 0.01.

While PD1/PD-L1 are upregulated in human and mouse tumors [22], including lung cancer cells (see Fig 1), the underlying mechanisms, particularly in the content of cisplatin resistance, remain elusive. As recent studies hint PD1/PD-L1 as potential targets of DNA methylation and/or RTK signaling [28–30], we initially measured the levels of DNMT1 and KIT in H69R and H82R cells. We provided the first evidence that both DNMT1 and KIT were upregulated in resistant versus parental cells at the levels of RNA and protein expression (Fig 3A). When DNMT1 or KIT was depleted by shRNA in H69R and H82R cells (Fig 3B), as expected, PD-L1 expression was significantly decreased (Fig 3C). To demonstrate the specific effects of DNMT1 or KIT on PD-L1 gene expression, we transfected H69R and H82R cells with DNMT1 or KIT shRNA vectors for 24 hours, and then introduced their expression vectors for additional 24 hours. Consistently, as shown in Fig 3D, overexpression of DNMT1 or KIT increased, whereas depletion of them decreased, PD-L1 expression. Importantly, PD-L1 downregulation by DNMT1 or KIT silencing was restored by DNMT1 or KIT re-introduction. These findings support the idea that DNMT1 or KIT functions as an upstream regulator of PD1/PD-L1 signaling in cisplatin resistance.

### Knockdown of DNMT1 or KIT sensitizes H69R and H82R cells to cisplatin treatment or PD-L1 depletion

**Fig 4:**
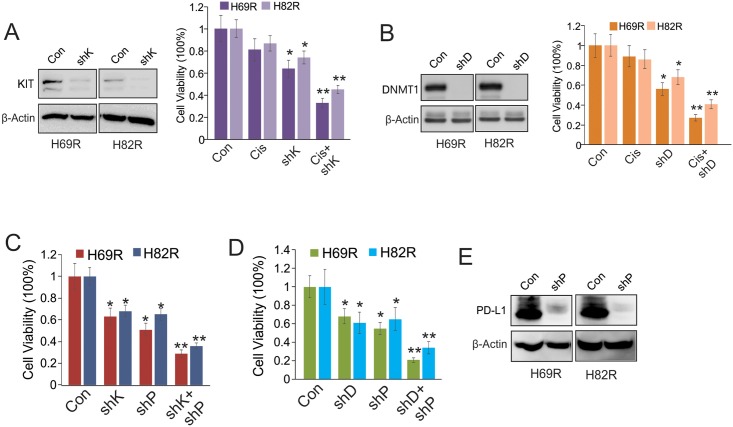
DNMT1 or KIT knockdown induces growth arrest in H69R and H82R cells. A and B, H69R and H82R cells were transfected with KIT (A) or DNMT1 (B) shRNA or control vectors for 24 hours, and then exposed to 3 μM of cisplatin for additional 24 hours. C and D, H69R and H82R cells were transfected with KIT (C) or DNMT1 (D) shRNA or control vectors for 24 hours, and then further transfected with PD-L1 shRNA vectors for additional 24 hours. E, The PD-L1 shRNA-transfected cells were subjected to Western blot analysis for PD-L1 expression. All the transfected cells were subjected to CCK8 assays; The gene knockdown was confirmed by Western blot; The experiments were done two times independently with 8 replicates; *P < 0.05, **P < 0.01. Note: Con, Control vectors; shP, PD-L1 shRNA; shD, DNMT1 shRNA; shK, KIT shRNA; Cis, Cisplatin.

The upregulation of DNMT1 or KIT in H69R and H82R raises the possibility that DNMT1 or KIT levels influence SCLC cell survival and proliferation in response to cisplatin. To test this, we initially knocked down DNMT1 or KIT itself in H69R and H82R cells, and found that the cell proliferation was significantly blocked (now shown). We then transfected the H69R and H82R cells with DNMT1 or KIT shRNA for 24 hours, exposed these cells to 3 μM cisplatin for another 24 hours, and evidenced that DNMT1 or KIT knockdown (Fig 4A and 4B, left panel) induced more pronounced cell growth inhibition in the presence of 3 μM cisplatin (Fig 4A and 4B, right panel), suggesting that abrogation of DNMT1 and KIT restores cisplatin sensitivity.

As H69R and H82R cells display upregulation of DNMT1, KIT and PD-L1, and given that disruption of DNMT1 or KIT partially impairs PD-L1 expression, the inhibitory effects of PD-L1 on resistant cell growth could be enhanced by DNMT1 or KIT depletion. To this end, we transfected H69R and H82R with DNMT1 or KIT shRNA vectors for 24 hours, and then introduced PD-L1 shRNA vectors into these cells for another 24 hours. As shown in Fig 4C and 4D, DNMT1 or KIT depletion (see Fig 4A and 4B, left panel) augmented the cell growth arrest mediated by PD-L1 knockdown (Fig 4E). These finding suggest that PD-L1 signaling could have a cooperative effect with DNMT1 or KIT activity on sustaining the growth of H69R and H82R.

## Discussion

It is well appreciated that resistance to cisplatin accounts for the therapeutic failure in treating SCLC patients, but the resistance mechanisms are largely unclear. Our findings identify PD1/PD-L1 signaling as a hitherto underappreciated defense mechanism for SCLC cells surviving through cisplatin selection. We demonstrate that upregulation of PD1/PD-L1 in cisplatin resistant cells might result from deregulation of DNMT1 and KIT, whose knockdown enhances the cell growth arrest mediated by the depletion of PD-L1. These finding support that a cooperative action among PD1/PD-L1, DNMT1 and KIT provides a unique proliferative advantage to SCLC cells in response to cisplatin.

It is a well-accepted concept that PD1 binds its ligand PD-L1 resulting in the protection of cancers from immune-mediated rejection [20, 21]. In accordance, antibody-based therapeutics targeting PD1/PD-L1 axis has entered into clinical trials for patients afflicted with immunogenic cancers [23]. PD1/PD-L1 are also overexpressed in human and mouse tumors, including NSCLC [25–27], and have cellular functions through activating mTOR/PI3/AKT signaling [22]. However, the influence of PD1/PD-L1 axis on SCLC cell survival and proliferation in context of cisplatin treatment remains unexplored. We have for the first time shown that PD1 and PD-L1 are expressed at a higher level in H69R and H82R compared to their respective parental cells. The facts that silencing of PD-L1 induces cell growth arrest and sensitizes H69R and H82R cells to cisplatin treatment support the notion that the cellular function of PD1/PD-L1 signaling is required to sustain survival and proliferation of cisplatin resistant cells. These findings provide a rationale for utilizing PD1/PD-L1 blocking antibodies as a single agent to cure refractory SCLC patient receiving cisplatin therapy.

The mechanisms underlying cisplatin resistance are complex and multiple stemming from multiple epigenetic and genetic changes [31]. In addition to PD1/PD-L1 signaling, we mainly examined the alterations of DNA methylation regulator DNMT1 and protein kinase KIT in current study. It is because that the development of cisplatin resistance [32, 33] and the alterations of DNA methylation/RTK signaling [34, 35] display similar traits: the promptness and reversibility. It is possible that upon exposure to cisplatin, the flexibility of DNA methylation/RTK signaling rapidly changes the transcriptome allowing certain cells to survive and proliferate through cisplatin-induced lethality. Second, our recent findings revealed that the deregulated DNMT1 and KIT significantly contributes to the resistance of molecular-targeted therapy [36]. Third, previous findings showed that many genes are hypermethylated [31, 37–39] and certain kinases are reactivated [40–43] in cisplatin resistance of NSCLC, ovarian and melanoma lung cancer. Fourth, ours and other findings revealed the overexpression of PD1/PD-L1 in human cancers, including NSCLC tumors, and cisplatin-resistant SCLC cells for largely unknown reasons. Recent investigations raise the possibility of PD1/PD-L1 as potential targets of DNA methylation and/or RTK signaling [28–30]. In accordance, our studies disclosed that DNMT1 and KIT were upregulated in H69R and H82R cells. Depletion of DNMT1 or KIT sensitized H69R and H82R to cisplatin, but knockdown of both led to more pronounced cell growth arrest. These discoveries demonstrate the crucial contribution of DNMT1 or KIT to cisplatin resistant phenotypes, supporting DNA hypomethylating agents and kinase inhibitors as therapeutic options in cisplatin-treated SCLC patients, which certainly merits further investigations. Moreover, we present evidence that KIT or DNMT1 abrogation reduced PD-L1 expression and enhanced cell growth arrest by PD-L1 ablation. These results identify KIT and DNMT1 as new upstream regulators of PD-L1 deregulation and support the existence of the DNMT1-KIT-PD-L1 cascade in cisplatin resistance, which requires the systematic and comprehensive characterization.

In sum, our current findings identify DNMT1-KIT-PD-L1 cascade as a unique resistance mechanism to cisplatin and provide insight into future therapeutic strategies for the treatment of cisplatin-refractory SCLC patients. These results are clinically appealing as combined treatment with an PD1/PD-L1 blocking agent and KIT or DNMT1 inhibitors, specifically in cisplatin refractory SCLC patients with evidence of deregulated DNMT1-KIT-PD-L1, may lead to a longer time to progression than is currently observed, and at the same time, increase the therapeutic index of these agents in SCLC management.

## Materials and Methods

### Plasmids and cell lines

The shRNA and scramble vectors for PD-L1, DNMT1 and KIT were obtained from BMGC RNAi (University of Minnesota). KIT expression plasmid was obtained by inserting KIT gene sequence into pBABE-puro retroviral vector. pCMV-Myc-DNMT1 expressing the Myc-tagged full-length DNMT1 was used. Cell lines, H1975, H1299, A549, H1650, H520, H460, H441, H358, H69, H82 and MRC-5, were obtained from American Type Culture Collection. Human normal lung fibroblast MRC-5 was cultured in EMEM and others in RPMI1640 supplemented with 50 μg/mL streptomycin, 50 IU/mL penicillin plus 10% fetal bovine serum (FBS) (Life Technology). Cisplatin was purchased from Sigma.

### In vitro adaption of cisplatin resistant cells

H69 and H82 cells were passaged with low concentrations of cisplatin (0.1 μM) and sequentially cultured in increasing concentrations of these TKIs (0.3, 1, 3 μM) for 6–8 weeks [36]. Cells cultured in parallel without drugs were served as parental sensitive controls. Cells were considered resistant when they could routinely grow in medium containing 3 μM of cisplatin.

### Transfections

The transfection of shRNA, expression or vehicle vectors was performed using Lipfectomine as previously described [36, 44, 45]. Briefly, H69R and H82R cells (2×10^5^) were seeded into 6-well plates overnight and then transfected with ~2.5 μg shRNA, expression or vehicle vectors using Lipofectamine^™^ 2000 reagent (Life Technologies, Carlsbad, CA). For co-transfection experiments, the total amount of the transfected DNA was reduced to half of that in the individual agent group, and kept consistent with the co-transfection group by adding vehicle vectors.

### Cell Counting Kit-8 (CCK-8) assays

After various treatments, the viable cells were counted by using the CCK-8 assay Kits (Dojindo Laboratories, Kumamoto, Japan) following the manufacturers’ instruction. Four wells were sampled per each experimental group in a given experiment. Averages were reported as ± Standard Deviation (SD).

### Western blotting

After various treatment, the cells were harvested in 1× protein lysis buffer [44, 45] and the Western blot using the whole protein lysates was performed as previously described [44, 45]. The antibodies used are: anti-DNMT1 (New England Biolabs, Ipswich, MA); anti-KIT and anti-PD-L1 (Cell Signaling Technology, Danvers, MA); anti-PD1 (Abcam, Cambridge, MA); anti-β-actin (Santa Cruz Biotechnology, Dallas, Texas).

### RNA isolation, cDNA preparation and qPCR

RNA was isolated using miRNAeasy Kit (Qiagen) and the first strand cDNA synthesis was carried out using the SuperScript^®^ III First-Strand Synthesis System (Invitrogen) [45, 46]. The qPCR was performed by SYBR-Green master mix, which was normalized by 18S levels [36, 44]. The primer sequences are:

PD-L1 forward: TCCACTCAATGCCTCAAT,

PD-L1 reverse: GAAGACCTCACAGACTCAA;

PD-1 forward: AAGTTTCAGGGAAGGTCAG,

PD-1 reverse: CTGGGCATGTGTAAAGGT;

DNMT1 forward: AGATGACGATGAGGAAGT,

DNMT1 reverse: ATGCGATTCTTGTTCTGT;

KIT forward: GCAAATACACGTGCACCAAC,

KIT reverse: GCACCCCTTGAGGGAATAAT;

18S forward: ACAGGATTGACAGATTGA,

18S reverse: TATCGGAATTAACCAG ACA.

### Lung cancer patients

The patients were diagnosed as NSCLC without smoking history. All the cases had matched normal tissues. The study protocols and the informed consent document signed by all patients before entering any study were approved by Mayo Clinic Institutional Review Board.

### Statistical analysis

The quantification for target changes was performed using the Student's t test. All statistical analysis was done using GraphPad Prism 5.0. Differences were considered statistically significant at *P* < 0.05. All P values were determined by unpaired, two-tailed Student’s t test.
